# Natural flowering control of pineapple ‘Pérola’ and ‘Vitória’ using aviglycine hydrochloride

**DOI:** 10.3389/fpls.2025.1578598

**Published:** 2025-07-02

**Authors:** Andrea Pires, Laís Fontana Silva, Thayanne Rangel Ferreira, Jeane Crasque, Basílio Cerri Neto, Lúcio de Oliveira Arantes, Edilson Romais Schmildt, José Aires Ventura, Vinicius de Souza Oliveira, Sara Dousseau-Arantes

**Affiliations:** ^1^ Departamento de Ciências Agrárias e Biológicas, Centro Universitário Norte do Espírito Santo, Universidade Federal do Espírito Santo, São Mateus, Espírito Santo, Brazil; ^2^ Departamento de Ciências Biológicas, Centro de Ciências Humanas e Naturais, Universidade Federal do Espírito Santo, Vitória, Espírito Santo, Brazil; ^3^ Centro de Pesquisa, Desenvolvimento e Inovação Norte, Instituto Capixaba de Pesquisa, Assistência Técnica e Extensão Rural, Linhares, Brazil

**Keywords:** *Ananas comosus* L., floral inhibition, flowering, growth regulators, management

## Abstract

**Introduction:**

Aviglycine hydrochloride (Aminoethoxyvinylglycine (AVG)) is an ethylene synthesis inhibitor recommended worldwide for controlling natural flowering of pineapple plants. However, the recommended dose depends on the environment, genotype and age of the plant. The objective was to evaluate the effect of AVG concentration in three application periods for controlling flowering in ‘Pérola’ (susceptible to fusariosis) and ‘Vitória’ (resistant to fusariosis) pineapple plants.

**Methods:**

The study was conducted in the field in the municipality of Sooretama, Espírito Santo-ES, Brazil. The experimental design used was randomized blocks in a 3×3+1 factorial scheme, consisting of three concentrations (100, 200 and 400 mg L^-1^) and three application times (April–July, May–July and June–July), with four replicates. The control was the plants without application. Flowering, phytotoxicity, morphological characteristics of plants, photosynthetic pigments, carbohydrate allocation and physical-chemical characteristics of fruits were evaluated.

**Results:**

AVG controlled natural flowering in both pineapple cultivars and the effect was proportional to the increase in concentration. Increasing the concentration and number of applications induced phytotoxic effects and reductions in starch and photosynthetic pigment contents in the leaves, also reducing vegetative development and fruit weight. The cultivar Pérola was more susceptible to natural flowering control, with a longer period of floral inhibition and reduction in vegetative and reproductive development when 400 mg L^-1^ was applied.

**Discussion:**

Therefore, we recommend that AVG be applied at a concentration of 100 mg L^-1^ before the climatic conditions for natural floral induction occur, which in the case of the northern coast of Espírito Santo, Brazil, occurs between June and July, and can extend until August, if nighttime temperatures are below 20 °C, being responsible for controlling natural flowering by 80%.

## 1 Introduction

The pineapple plant (*Ananas comosus* var. comosus) is one of the main fruit species and Brazil is the fifth largest producer in the world, with 2,387,393 tons of fruit produced in 2023 (FAO, 2024). Despite the notorious importance of pineapple in the Brazilian production scenario, the average productive yield is quite variable, producing between 7,756 and 33,000 kg ha^-1^ (IBGE, 2024). This fact is mainly associated with the fusariosis disease (*Fusarium subglutinans*) and the technological level applied to the crops, where nutritional and water management are deficient and natural flowering control is not used to scale production, only artificial floral induction is carried out to standardize flowering (Galeano et al., 2022).

Floral induction in pineapple occurs as a result of short days and cold nights (Wang and Paull, 2018), and a difference of less than 4°C between the minimum and maximum temperatures of the day is related to a high incidence of natural floral induction (Jiménez and Villalobos, 2019). The natural flowering of pineapple occurs under normal conditions between late autumn and early winter, which in the southern hemisphere corresponds to the months of June to August. This condition results in uneven fruiting and harvesting, with increased harvesting costs and greater difficulties with cultural treatments (Maia et al., 2019), in addition to irregularity in the supply of fruits throughout the year (Bartholomew, 2014), which makes the agronomic management of the crop difficult.

Furthermore, pineapple is considered a non-climacteric fruit, that is, it only ripens attached to the mother plant, thus not having the ability to ripen post-harvest (Santos and Borém, 2019). This fact means that pineapple fruits have to be harvested close to the ideal ripening point, which often results in fruits with different ripening stages caused by irregularity when the plants are in natural flowering. In the vast majority of cases, fruits have physiological responses linked to ethylene biosynthesis during the ripening process that modify their texture, color, aroma, and flavor (Handa et al., 2012). However, for non-climacteric fruits, such as pineapple, ripening occurs independently of ethylene (Taiz et al., 2022). Thus, the fruit ripening process is not affected by the inhibition of ethylene synthesis.

The perception of these factors that induce flowering induces changes in hormonal balance, and ethylene is the main hormone involved (Espinosa et al., 2017). Knowledge of the ethylene biosynthesis pathway has enabled the emergence of commercial compounds that can be sprayed on the plant and act by inhibiting or stimulating its endogenous production, enabling the management of pineapple flowering. Among these compounds is aminoethoxyvinylglycine (AVG) (Khan and Ali, 2018), marketed in Brazil with the aim of increasing fruit retention in apple trees (*Malus domestica* Borkh.).

The floral inhibition effect caused by AVG is associated with its action in blocking ACC synthase, which is responsible for initiating ethylene synthesis (Schaller and Binder, 2017). AVG inhibits the activity of enzymes dependent on the pyroxidal phosphate group, including ACC synthase, an enzyme that catalyzes the conversion of S-adenosyl methionine to ACC (Van De Poel and Van Der Straeten, 2014). Over time, AVG is metabolized, new synthesis of ACC synthase occurs, and the plant recovers its ability to synthesize ethylene. Therefore, it is important to evaluate the concentration and frequency of application according to the induction period characteristic of each producing region.

Products containing AVG are recommended worldwide for inhibiting the natural flowering of pineapple plants. However, the recommended dose depends on the environment, genotype and age of the plant, and if not applied correctly, may increase production costs and generate monetary losses. Therefore, with the aim of supporting production/harvesting strategies for high-quality pineapple fruits and during periods of better fruit prices, the effect of the dose and period of control of AVG on ‘Pérola’ and ‘Vitória’ pineapple plants was evaluated.

## 2 Materials and methods

**Figure 1:**
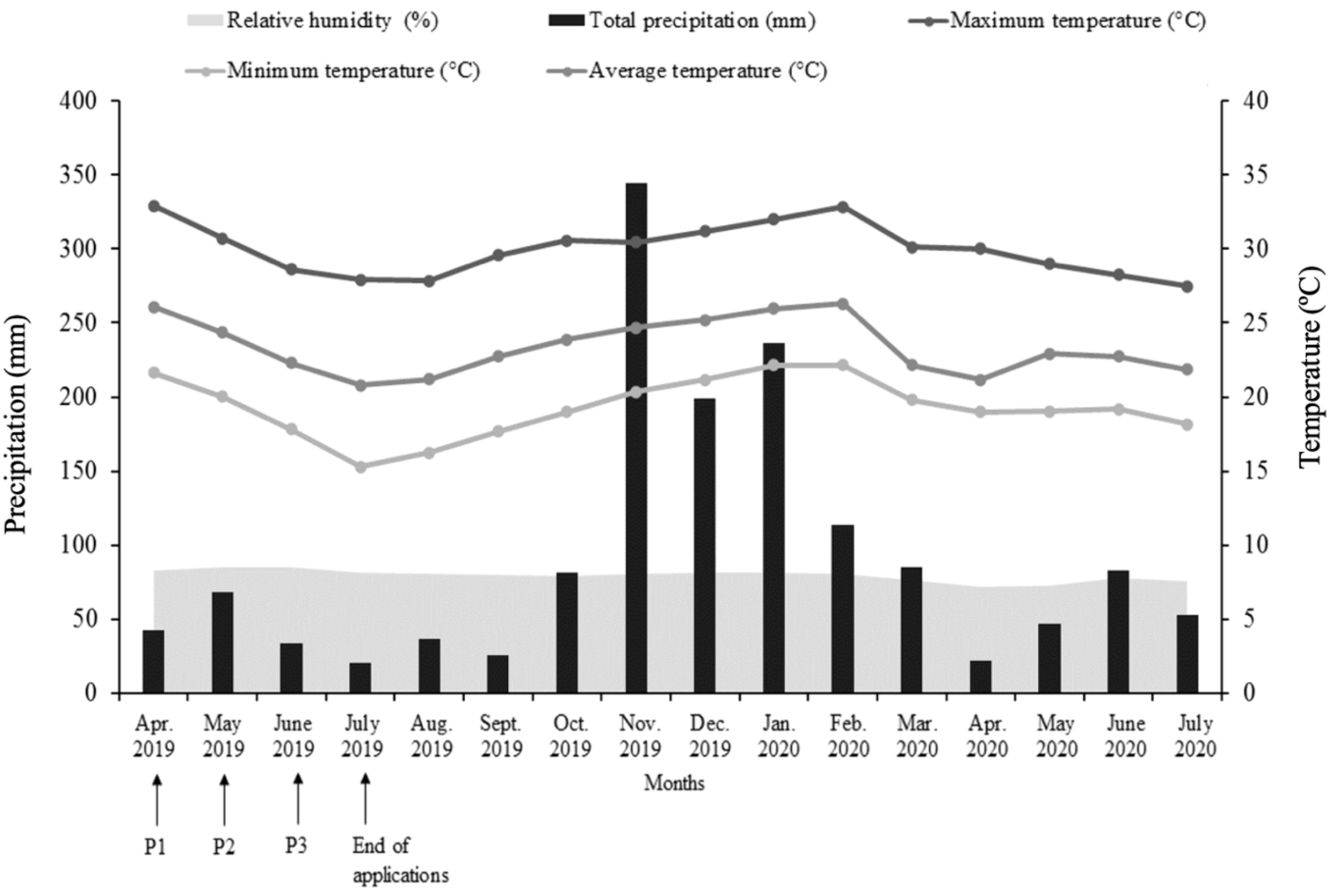
Total precipitation (mm), relative humidity (%), and maximum, average and minimum air temperatures recorded at the weather station of Sooretama and Linhares both in the state of Espirito Santo, from January 2019 to July 2020. Application period: P1 (April-July), P2 (May-July), and P3 (June-July). Source: Incaper, 2020.

The study was carried out from March 2019 to April 2020 and from May to July 2020, at the Experimental Farm of the Capixaba Institute for Research, Technical Assistance and Rural Extension (INCAPER), located in the municipality of Sooretama/ES (latitude 19°11’30” S, longitude: 40°05’44” W and altitude of 30 meters). The climate of the region is tropical Aw, with rain in summer and dry winter, according to the Köppen classification (Alvares et al., 2014). The monthly data on minimum, maximum and average temperatures (°C), precipitation (mm) and relative humidity (%) during the experimental period, obtained through the automatic Meteorological Station (INCAPER, 2020) are presented in **Figure 1**.

Seedlings of the pup type with an average mass between 100-200 g were used. For the cultivar Pérola, the seedlings were purchased from producers in the region of Marataízes/ES, while those of the cultivar Vitória were obtained from the Incaper Experimental Farm in Pacotuba/ES. The seedlings were immersed for three minutes in a solution containing the insecticide thiamethoxam (250 g kg^-1^) from the neonicotinoid chemical group, for the disinfestation of the mealybug (*Dysmicoccus brevipes* C.). In the case of cv. Pérola, the seedlings were also immersed in the fungicide thiophanate methyl (700 g kg^-1^), to prevent fusarium wilt. Subsequently, the seedlings were dried in the shade for three days and planted in the field in the second half of April 2018.

The planting system adopted was in double rows with a spacing of 0.9 x 0.4 x 0.30 m, under black mulching and maintained under drip irrigation (Oliveira et al., 2021). Fertilization was carried out based on the results of the soil analysis and as indicated in the fertilization and liming manual for the state of Espírito Santo (Prezotti et al., 2007). The soil analysis showed the following results: pH 4.92, P 3.52 mg/dm^3^, K 30 mg/dm^3^, Ca 0.71 cmolc/dm^3^, Mg 0.20 cmolc/dm^3^, H + Al 3.36 cmolc/dm^3^, S 0.99 cmolc/dm^3^, T 4.35 cmolc/dm^3^, t 1.09 cmolc/dm^3^, m 9.21%, V 23%, MO 4.72 dag/dm^3^, Bo 0.09 mg/dm^3^, Cu 1.1mg/dm^3^, S 4.67 mg/dm^3^, Fe 136mg/dm^3^, Mn 7.8 mg/dm^3^, Na 63.7 mg/dm^3^, Zn 3.7 mg/dm^3^. During the cultivation of the plants, the supply of nitrogen and potassium was adjusted for the crop, in a liquid form, through localized fertigation and with the application of 16 equal doses at decreasing time intervals, according to Souza and Almeida (2002).

The experiments conducted with each cultivar were considered independent and maintained in a randomized block design in a factorial scheme (3x3 + 1) where the first factor consisted of three concentrations (100, 200 and 400 mg L^-1^) of aviglycine hydrochloride (Retain^®^ TM 15% i. a.) and the second, of application periods with weekly interval (16, 12 and 8 applications) started in April (02/04/2019-16/07/2019), May (07/05/2019-23/07/2019) and June (03/06/2019-23/07/2019), respectively. 0.05% of the nonionic silicone adhesive spreader (Silwet) was used. Plants without spraying were used as an additional control. Each treatment consisted of four replicates and each plot consisted of 24 plants. Spraying was carried out in the early hours of the day, using a manual backpack sprayer with a capacity of 20 liters and with a jet directed towards the central leaf rosette (50 mL^-1^).

Natural flowering inhibition data were obtained by counting plants with visible inflorescence in the leaf rosette, carried out weekly from July 2019, the period in which the additional control began flowering. Artificial floral induction was performed 20 months after planting, in December 2019, when stabilization of flowering in AVG treatments was observed. An ethephon-based product was used, with 240 g L^-1^ of ethephon (200 mL of Ethrel 100 L^-1^ of water + 2 kg of urea) (Antunes et al., 2008), in all plants that did not present visible inflorescence in the leaf rosette. Flowering evaluations were completed in the second half of February 2020, when flowering of the induced plants was no longer observed. Based on the data obtained, the accumulated flowering over time was calculated, expressed as a percentage.

**Figure 2:**
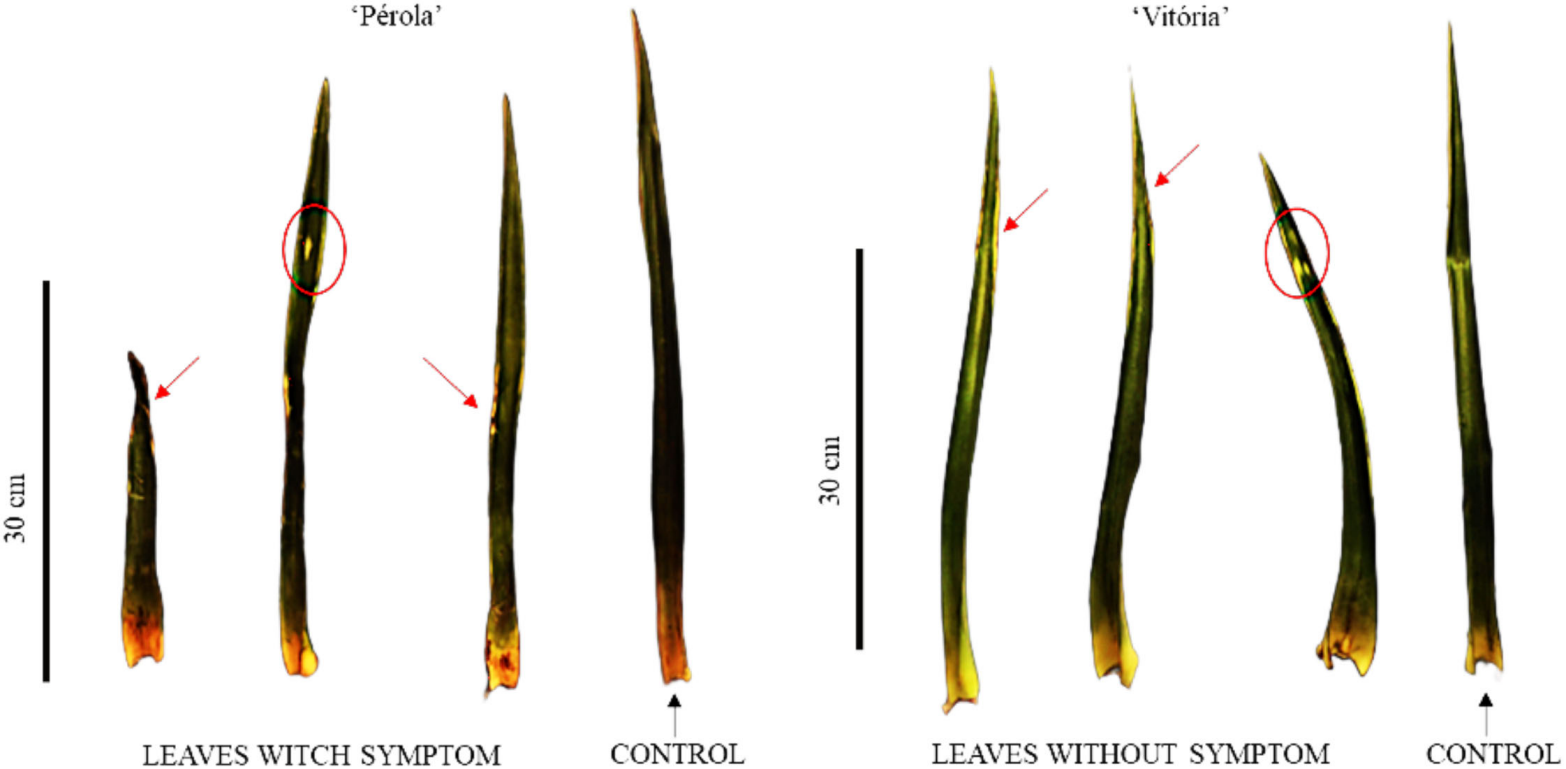
Criteria used to determine leaves with and without phytotoxicity symptoms in ‘Pérola’ and ‘Vitória’ pineapple plants at artificial floral induction in plants sprayed with different concentrations of Aviglycine hydrochloride (AVG) and application frequencies.

In artificial floral induction, two plants were collected per plot and morphological characterization was performed. The height from the base to the apex of the tallest leaf of the plant, expressed in cm, was evaluated; length of leaf ‘D’, expressed in cm; total number of leaves; and number of leaves with and without symptoms of phytotoxicity, counted manually. Leaves with and without symptoms of phytotoxicity were those that showed signs of chlorosis or necrosis (**Figure 2**). The number of leaves with symptoms was used to determine the percentage of leaves with phytotoxicity.

Photosynthetic pigments were extracted from fully expanded leaf D. Chlorophylls were extracted using the methodologies proposed by Arnon (1949). For carotenoids, the methodology of Rodriguez-Amaya and Kimura (2004) was used. For both chlorophylls and carotenoids, 80% acetone was used as the extracting solution. A 2 mm diameter disc of the leaf was used, weighed and macerated. Subsequently, 10 mL of the extracting solution was added. The mixture remained for 12 h in a container without light and covered. After this period, the content was filtered through paper and read in a spectrophotometer (Beckman, model 640B) at 645 and 663 nm for chlorophylls and 450 nm for carotenoids. To calculate the chlorophylls, the values ​​found in absorbance (A) were substituted in Equations 1, 2, 3. To calculate the concentration of total carotenoids, Equation 4 was obtained, using the values ​​in absorbance (A) (450 nm), volume of the container used in the analyses (V in mL), mass of the sample (m in g) and extinction coefficient ( $\text{E}\frac{1\%}{1\text{cm}}$ = 2,592). The results of the concentration of chlorophyll a, b, total and carotenoids were expressed in µg.g^-1^ of fresh leaf mass of plants.


(1) Chlorophyll a=12.7×A(663)−2.69×A (645)  

(2) Chlorophyll b=22.9×A(645)−4.68×A (663)

(3) Chlorophyll Total=20.2×A(645)+8.02×A (663)

(4) $$\text{Total carotenoids}=\frac{\text{A}\times \text{V}\times 10^{4}}{\text{E}\frac{1\%}{1\text{cm}}\times \text{m}}$$

Carbohydrate allocation was evaluated by quantifying reducing sugars (RS) and total soluble sugars (TSS), and starch in leaves with and without symptoms of phytotoxicity. The plant tissues used to evaluate dry mass were ground in a Willey knife mill, model STAR FT-50, and stored in a freezer at -18°C. The extracts were obtained according to Zanandrea et al. (2009), using a mass of 0.2 g. The Anthrone method (Yemm and Willis, 1954) was used to quantify TSS, with modifications, using 2 mL of a 0.19% anthrone solution in 93.33% sulfuric acid in a reaction volume of 3 mL, subjected to 100°C for 3 minutes. RS were quantified according to the protocol described by Miller (1959), using the Dinitrosalicylic Acid (DNS) method. The results were expressed in mg.glucose g^-1^ of leaf dry mass.

The fruit harvest began in November 2019, when 11 to 25% of the peel was yellow-orange, a ripening stage called painted, according to Normative Instruction/SARC No. 001 for white-fleshed pineapple (MAPA, 2020). The fruits were evaluated individually and the fruit mass was determined by weighing them individually on a semi-analytical precision electronic scale, Marconi model, No. AS5500C. The fruits were peeled manually and the juice was extracted in a Philips Walita BR Juicer 700W centrifuge to quantify Soluble Solids (SS) (°Brix), Titratable Acidity (TA) (% citric acid), and Soluble Solids/Titratable Acidity ratio (Ratio).

The SS levels were obtained from a 1 mL aliquot of juice using a Schmidt Haensch ATR-BR^®^ digital benchtop refractometer, ranging from 0 to 100°Brix. The AT was performed using the 0.1 N NaOH method in a Titrino Plus Metrohn/848 automatic titrator, obtaining the result in % of citric acid present. The ratio was determined by the ratio between the two variables (SS/AT).

The data were subjected to analysis of variance and Tukey test at 5% probability using the statistical software R, version 4.0.2, R Studio 3.0.1 (R Core Team, 2024) and the ExpDes.pt package (Ferreira et al., 2018).

## 3 Results

**Figure 3:**
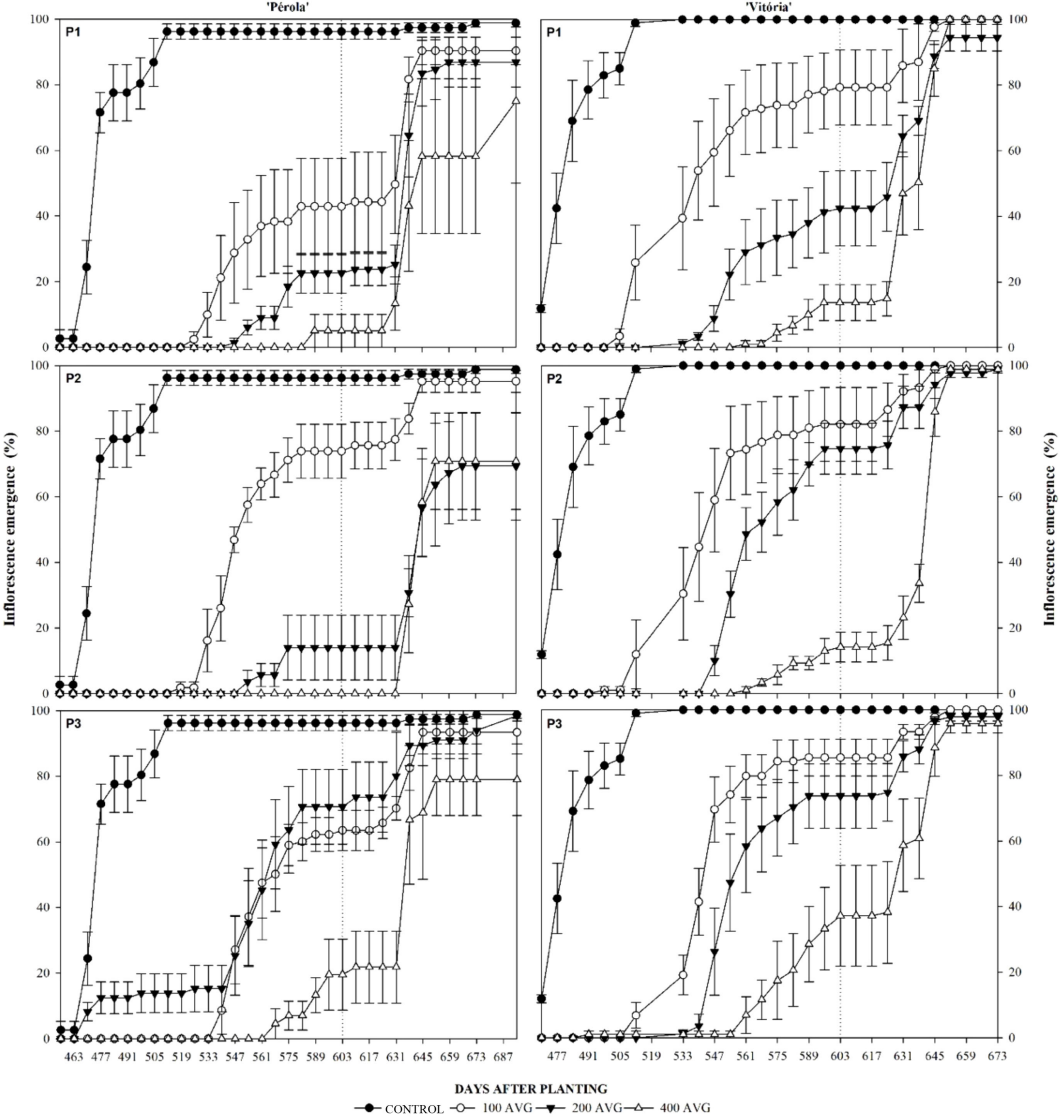
Cumulative percentage of inflorescence emergence on ‘Pérola’ and ‘Vitória’ pineapple plants sprayed with Aviglycine Hydrochloride at concentrations of 100, 200, and 400 mg L^-1^ (100, 200, 400) + additional control (TEST) in Period 1 (April 02, 2019-July, 16 2019), Period 2 (May, 07 2019-July 23, 2019) and Period 3 (June 03, 2019-July 23, 2019). The dotted line indicates the date of plant induction (IND) (December 12, 2019). The bar corresponds to the standard error of the mean of four replicates of 24 plants per plot.

Visualization of the inflorescence emergence of pineapples of cultivars Vitória and Pérola started in July at 470 DAP and reached over 80% in August, at 498 DAP (**Figure 3**). The flowering of the Pérola and Vitória cultivars was inhibited by AVG and an increase in the inhibition time was observed as the concentration was increased (**Figure 3**). The Pérola cultivar was more susceptible to the control of natural flowering, with a longer period of floral inhibition, varying between three and four weeks longer than the Vitória cultivar, for most AVG concentrations.

The cultivar Pérola, when subjected to a concentration of 100 mg L^-1^, showed a flowering completely inhibited for nine to eleven weeks, in the treatment with 200 mg L^-1^ for 12-13 weeks and in that of 400 mg L^-1^ for 15-18 weeks (**Figure 3**). Regarding cultivar Vitória, when subjected to a concentration of 100 mg L^-1^, flowering was inhibited for three to five weeks, at a concentration of 200 mg L^-1^ for seven to ten weeks, and at a concentration of 400 mg L^-1^ for 13-14 weeks. Among the application frequencies, a variation of a maximum of three weeks of inhibition was found with a very similar response. The exception is only for the Pérola cultivar at a concentration of 400 mg L^-1^ applied in P2 (May to July) whose inhibition time increased to 24 weeks and at a concentration of 200 mg L^-1^ applied in P3 (June to July) that had control of only one week.

In December, an stabilization was observed in the flowering of the plants treated with AVG, when we performed the artificial floral induction (December 09, 2019), to verify if the plants remained responsive and if there would be any reflection on fruit production. On this date, plants sprayed with AVG maintained significantly lower inhibition compared to non-sprayed plants, regardless of the cultivar (**Table 1**). The accumulated flowering of the Pérola cultivar was 21.17% lower than that of the Vitória cultivar, showing that the greater sensitivity of this cultivar to AVG was maintained over time.

**Table 1:** Percentage of inflorescences in artificial floral induction in ‘Pérola’ and ‘Vitória’ pineapple plants sprayed with Aviglycine Hydrochloride (AVG) at concentrations of 100, 200, and 400 mg L^-1^ (100, 200, 400) + control, in Period 1 (P1) (April 02 2019-July 162019), Period 2 (P2) (May 07 2019-July 23 2019) and Period 3 (P3) (June 03 2019-July 23, 2019) to 603 days after flowering (September 12, 2019).

AVG	‘Pérola’				‘Vitória’			
	Application			Mean	Application			Mean
	P1	P2	P3		P1	P2	P3	
100	42.92 aB	73.88 aA	63.51 aAB	60.10	79.29	82,07	85.42	82.26 a
200	22.56 abB	13.99 bB	70.72 aA	35.75	42.44	74.64	73.71	63.60 a
400	5.00 bA	0.00 bA	19.55 bA	8.18	13.66	14.17	37.20	21.68 b
Means	23.49	29.29	51.25		45.13 B	56.96 AB	65.44 A	
Control				97.42 a				100.0 a
Factorial				34.68 b				55.85 b

Means followed by the same lowercase letters in the columns and uppercase letters in the rows do not differ from each other according to the Tukey test (p<0.05).

Regardless of the cultivar, we found that spraying that started in P3 (June to July) had a significant reduction in flowering control (**Table 1**). The cultivar Pérola showed an interaction between concentrations and periods of application of AVG, with a lower percentage of flowering when higher concentrations were used in P1 and P2. The cultivar Vitória was influenced by concentrations and application periods, with lower percentages at concentrations 400 and P1.

After the effect of inhibiting natural flowering, the cultivars showed a distinct response to artificial flowering induction (**Figure 3**), with values of flowering recovery above 90% for cultivar Vitória, regardless of concentration and period. The cultivar Pérola sprayed at a concentration of 400 mg L^-1^ showed flowering below 80%, regardless of the frequency of application.

Phytotoxic effects were observed in plants sprayed with AVG, characterized by leaf chlorosis, which in some cases evolved to necrosis (**Table 2**). The cultivar Pérola was more sensitive to the effect of the concentrations, with a higher percentage of leaves with symptoms even at the lowest concentrations, but with approximately 40% of leaves with symptoms at the concentration of 400 mg L^-1^, a value similar to that of the cultivar Vitória. In both cultivars, the degree of the symptom was influenced by the concentrations and application periods, with an increase in the percentage of leaves with symptoms as the concentration was increased and when a greater number of applications were performed, from 12 to 16, respectively, periods 2 and 1.

**Table 2:** Percentage of leaves with phytotoxicity symptoms in ‘Pérola’ and ‘Vitória’ pineapple plants sprayed with Aviglycine Hydrochloride (AVG) at concentrations of 100, 200, and 400 mg L^-1^ (100, 200, 400) + control, in Period 1 (P1) (April, 02 2019-July 16, 2019), Period 2 (P2) (May 07, 2019-July 23, 2019) and Period 3 (P3) (June 03, 2019-July 23, 2019).

AVG	‘Pérola’				‘Vitória’			
	Application			Mean	Application			Mean
	P1	P2	P3		P1	P2	P3	
Leaves with symptoms (%)								
100	32,67	28.61	18.55	26.84 b	23.18	20.54	11.38	18.25 c
200	40.33	39.67	27.16	35.47 ab	32.89	29.77	23.09	28.74 b
400	41.55	40.43	33.51	38.47 a	46.92	40.64	32.76	40.38 a
Mean	38.18 A	36.23 AB	26.41 B		34.33 A	30.32 A	22.41 B	
Control				0.00%				0.00%
Factorial				35.11%				29.19%

Means followed by the same lowercase letters in the columns and uppercase letters in the rows do not differ from each other according to the Tukey test (p<0.05).

Vegetative development was also affected by the increase in AVG concentrations, and the concentration of 400 mg L^-1^ caused a greater reduction in plant height, regardless of the cultivar (**Table 3**). In the Pérola cultivar, the reduction observed between the concentrations of 100 mg L^-1^ and 400 mg L^-1^ was 10.96%, and in the Vitória cultivar, the reduction was 21.20%. The number of leaves was approximately 10% lower in plants treated with AVG, but it was not possible to identify differences between the concentrations and control periods. The length of the ‘D’ leaf showed a reduction of 6.23% and 11.39% in the Pérola and Vitória cultivars, respectively, when sprayed with AVG. The reduction was significant in plants that received a greater number of sprays (P1).

**Table 3:** Height (cm), ‘D’ leaf length (cm) and total number of leaves of ‘Pérola’ and ‘Vitória’ pineapple plants sprayed with Aviglycine Hydrochloride (AVG) at concentrations of 100, 200, and 400 mg L^-1^ (100, 200, 400) + control, in Period 1 (P1) (April, 2 2019-July16, 2019), Period 2 (P2) (May 07, 2019-July 23/2019) and Period 3 (P3) (June 03, 2019-July 23, 2019).

AVG	‘Pérola’				‘Vitória’			
	Application			Mean	Application			Mean
	P1	P2	P3		P1	P2	P3	
Height (cm)								
100	94.16	106.67	95.67	98.83 ab	101.17	105.33	102.33	102.94 a
200	100.50	97.50	104.83	100.94 a	86.00	95.83	96.33	92.72 ab
400	95.67	81.33	87.00	88.00 b	81.33	81.50	80.50	81.11 b
Mean	96.78 A	95.17 A	95.83 A		89.50 A	94.22 A	93.05 A	
Control				94.00 a				87.83 a
Factorial				95.93 a				92.26 a
‘D’ leaf length (cm)								
100	77.85 aB	91.78 aA	91.66 aA		63.81	69.52	81.25	73.43 a
200	77.62 aB	82.04 aB	99.74 aA		68.37	75.14	85.04	74.77 a
400	87.53 aAB	81.58 aB	95.03 aA		65.00	73.64	87.97	73.38 a
Mean					65.65 C	73.56 B	82.37 A	
Control				93.00 a				83.35 a
Factorial				87.20 a				73.86 b
Total number of leaves								
100	37.18	43.38	40.04	40.20 a	89.17	99	96.67	94.94 a
200	40.00	39.14	43.92	41.02 a	99.33	101.33	91.67	97.44 a
400	37.38	31.85	42.57	37.26 a	97.33	98.83	87	94.38 a
Mean	38.18 A	38.12 A	42.18 A		95.27 A	99.72 A	91.78 A	
Control				47.00 a				83.00 a
Factorial				39.50 b				95.59 b

Means followed by the same lowercase letters in the columns and uppercase letters in the rows do not differ from each other according to the Tukey test (p<0.05).

The increase in AVG concentration led to a reduction in chlorophyll a and b levels in plants sprayed with AVG, but for the Pérola cultivar there were no significant differences (**Table 4**). Total carotenoid levels were influenced only by the application periods, with a reduction in P3 (8 AVG applications) for the Vitória cultivar.

**Table 4:** Chlorophyll *a* (µg.g^-1^), chlorophyll *b* (µg.g^-1^), total chlorophylls (µg.g^-1^) and total carotenoids (µg.g^-1^) of ‘Pérola’ and ‘Vitória’ pineapple plants sprayed with Aviglycine Hydrochloride (AVG) at the concentrations of 100, 200, and 400 mg L^-1^ (100, 200, 400) + control, in Period 1 (P1) (April 02, 2019-July 16, 2019), Period 2 (P2) (May 07, 2019-July 23, 2019) and Period 3 (P3) (June 06, 2019-July 23, 2019).

AVG	‘Pérola’				‘Vitória’			
	Application			Mean	Application			Mean
	P1	P2	P3		P1	P2	P3	
Chlorophyll a (µg.g^-1^)								
100	17.17	23.95	20.8	20.16 a	13.74	10.46	10.56	12.18 ab
200	25.21	29.13	20.76	24.75 a	13.37	15.20	5.67	13.04 a
400	18.1	21.81	10.49	17.35 a	10.46	15.20	8.88	7.17 b
Means	20.42 A	25.03 A	16.80 A		9.52 A	11.36 A	11.51 A	
Control				24.10 a				16.72 a
Factorial				20.75 a				10.80 b
Chlorophyll b (µg.g^-1^)								
100	36.91	53.21	41.32	43.81 a	27.23	23.08	12.88	27.44 ab
200	50.33	62.51	46.53	53.12 a	30.75	31.01	16.34	29.50 a
400	45.07	44.81	22.86	37.58 a	24.35	34.41	20.89	16.70 b
Means	44.10 A	53.51 A	36.91 A		21.07 A	26.04 A	26.55 A	
Control				53.01 a				39.65 a
Factorial				44.84 a				24.55 b
Total chlorophylls (µg.g^-1^)								
100	54.08	78.43	59.42	63.95 a	39.57	44.49	34.81	39.60 ab
200	73.62	91.64	68.35	77.84 a	33.65	44.39	49.61	42.53 a
400	65.87	65.57	33.36	54.91 a	18.56	23.31	29.78	23.87 b
Means	64.50 A	78.52 A	53.69 A		30.58 A	37.38 A	38.05 A	
Control				77.09 a				56.35 a
Factorial				65.57 a				35.34 b
Total carotenoids (µg.g^-1^)								
100	94.78	74.63	96.55	88.65 a	78.09	94.91	72.75	81.92 a
200	98.44	87.61	69.04	85.03 a	97.40	116.27	72.53	95.40 a
400	95.9	119.86	65.78	93.84 a	99.70	97.41	83.45	93.52 a
Means	96.37 A	94.03 A	77.12 A		91.73 AB	102.87 A	76.24 B	
Control				99.40 a				71.37 a
Factorial				89.17 a				90.28 a

Means followed by the same lowercase letters in the columns and uppercase letters in the rows do not differ from each other according to the Tukey test (p<0.05).

Carbohydrate contents varied according to AVG concentrations and application periods, but with differences between cultivars (**Table 5**). Plants of the Vitória cultivar, sprayed with AVG, had lower reducing sugar contents in the leaves. Total soluble sugar contents were lower only in the Pérola cultivar, when subjected to 400 mg L^-1^ of AVG, regardless of the period. In the Vitória cultivar, plants sprayed with AVG showed a decrease in starch contents, more expressively at the concentration of 400 mg L^-1^.

**Table 5:** Reducing sugars (mg.glucose g^-1^), total soluble sugars (mg.glucose g^-1^) and starch (mg.glucose g^-1^) in the leaves of ‘Pérola’ and ‘Vitória’ pineapple plants sprayed with Aviglycine Hydrochloride (AVG) at the concentrations of 100, 200, and 400 mg L^-1^ (100, 200, 400) + control, in Period 1 (P1) (April 02, 2019-July 16, 2019), Period 2 (P2) (May 07, 2019-July 23, 2019) and Period 3 (P3) (June 06, 2019-July 23, 2019).

AVG	‘Pérola’				‘Vitória’			
	Application			Mean	Application			Mean
	P1	P2	P3		P1	P2	P3	
Reducing sugars (mg.glucose g^-1^)								
100	102.38	109.66	122.19	111.41 a	127.71 Aa	126.95 Aa	115.99 Aa	123.55a
200	124.75	97.12	107.18	109.68 a	101.66 Ab	126.09 Aab	136.78 Aa	121.51a
400	86.09	107.25	96.45	96.59 a	98.69 Ab	109.03 Aab	132.69 Aa	113.46a
Mean	104.40 A	104.67 A	108.60 A		109.35 A	120.69A	128.48 A	
Control				106.29 a				154.49 a
Factorial				105.90 a				119.51 b
Total soluble sugars (mg.glucose g^-1^)								
100	197.33	207.88	209.81	205.01 a	285.86	258.05	248.97	264.30 a
200	206.41	178.76	227.48	204.22 a	198.71	259.53	262.30	240.18 a
400	151.23	155.31	179.28	161.94 b	243.33	257.27	282.00	260.87 a
Mean	184.99 A	180.65 A	205.52 A		242.63 A	258.29 A	264.42 A	
Control				184.60 a				309.90 a
Factorial				190.38 a				255.11 a
Starch (mg.glucose g^-1^)								
100	117.73	104.09	107.20	109.67 a	127.85	119.55	114.68	120.69 a
200	101.53	83.91	107.89	97.78 a	107.30	113.46	106.60	109.11 ab
400	109.02	85.37	113.32	102.57 a	89.62	101.25	107.03	99.30 b
Mean	109.43 A	91.12 A	109.47 A		108.26 A	111.42 A	109.43 A	
Control				97.43 a				160.83a
Factorial				103.34 a				109.70b

Means followed by the same lowercase letters in the columns and uppercase letters in the rows do not differ from each other according to the Tukey test (p<0.05).

The physicochemical quality of pineapples from both cultivars evaluated was affected by AVG concentrations, but not by the frequency of application (**Table 6**). Plants subjected to AVG application showed a reduction in fruit mass with increasing concentration in both cultivars, but ‘Vitória’ reduced by 30.96% and Pérola by 15.26%. The Pérola cultivar at a concentration of 100 mg L^-1^ had a mass of 1.214 g, at 200 mg L^-1^ of 952.99 g and at 400 mg L^-1^ of 836.81 g. The Vitória cultivar at a concentration of 100 mg L^-1^ had a mass of 1165.92 g, at 200 mg L^-1^ of 1000.63 g and at 400 mg L^-1^ of 874.55 g. It was found that the Pérola cultivar was most affected by the concentrations, with a reduction of 21.58% between the concentration of 100-200 mg L^-1^, and of 12.10% between 200-400 mg L^-1^. In the Vitória cultivar, the reduction was 14.18% between the concentration of 100-200 mg L^-1^ and of 12.60% between 200-400 mg L^-1^.

**Table 6:** Mean values of fruit mass (g), soluble solids (°Brix), titratable acidity (% acid citric), ratio (SS/TA), and vitamin C (mg 100g^-1^) of ‘Pérola’ and Vitória pineapple plants sprayed with Aviglycine hydrochloride (AVG) at the concentrations of 100, 200 and 400 mg L^-1^ (100, 200, 400) + control, in Period 1 (P1) (April 02, 2019-July 16, 2019), Period 2 (P2) (May 07, 2019-July 23, 2019) and Period 3 (P3) (June 06, 2019-July 23, 2019).

AVG	‘Pérola’				‘Vitória’			
	Application periods			Mean	Application periods			Mean
	P1	P2	P3		P1	P2	P3	
Fruit mass (g)								
100	1,206.33	1,259.14	1,200.53	1,214.0 a	1,237.76	1,156.48	1,122.77	1,165.92 a
200	1,031.13	907.21	965.67	952.99 b	967.39	1,001.58	1,034.50	1,000.63 b
400	821.84	748.22	940.37	836.81 b	850.72	847.61	932.82	874.55 c
Mean	1,019.77 A	963.93 A	1020.51 A		1,017.00 A	1,001.89 A	1,022.2 A	
Control				1,181.74 a				1,468.28 a
Factorial				1,001.40 b				1,013.70 b
Soluble Solids (°Brix)								
100	12.89	12.37	12.42	12.51 a	13.84	14.24	14.39	14.23 b
200	13.11	12.26	11.86	12.40 a	15.68	14.75	14.52	14.98 ab
400	12.60	12.54	12.73	12.62 a	15.06	15.44	15.19	15.60 a
Mean	12.87 A	12.34 A	12.34 A		14.86 A	14.81 A	14.70 A	
Control				11.09 b				14.27 a
Factorial				12.52 a				14.79 a
Titratable acidity (% citric acid)								
100	0.51	0.51	0.6	0.52 c	0.65	0.66	0.6	0.63 a
200	0.54	0.56	0.63	0.57 b	0.69	0.64	0.62	0.65 a
400	0.63	0.66	0.61	0.63 a	0.63	0.69	0.66	0.66 a
Mean	0.59 A	0.57 A	0.56 A		0.66 A	0.66 A	0.63 A	
Control				0.50 b				0.65 a
Factorial				0.57 a				0.65 a
Ratio (SS/TA)								
100	23.78	23.96	24.54	24.6 a	22.6	22.91	25.2	23.57 a
200	22.34	22.92	22.11	22.46 ab	23.74	23.68	24.6	24.01 a
400	20.37	19.5	22.18	20.69 b	24.48	23.1	23.53	23.70 a
Mean	22.16 A	22.64 A	22.94 A		23.60 A	23.23 A	24.44 A	
Control				23.23 a				23.06 a
Factorial				22.58 a				23.75 a

Means followed by the same lowercase letters in the columns and uppercase letters in the rows do not differ from each other according to the Tukey test (p<0.05).

The chemical quality characteristics of the pineapples produced were affected by the AVG concentrations, but not by the period, with variations between the cultivars. In the Pérola cultivar, the AVG promoted an increase in the content of soluble solids and in the titratable acidity, producing more acidic pineapples with a lower Ratio with the increase in the applied concentration. For the ‘Vitória’ cultivar, the higher AVG concentrations provided an increase in the content of soluble solids.

## 4 Discussion

According to Zhang et al. (2016), the perception of inductive factors occurs approximately 40 days before the emergence of the inflorescence is observed, which allows us to conclude that the natural floral induction of most plants occurred during the months of June and July. During this period, we can observe the presence of inductive factors, long nights (short days) and minimum temperatures below 20°C (**Figure 1**), as observed for other cultivars, such as ‘MD-2’ (Jiménez and Villalobos, 2019).

The vegetative phase of the pineapple plant lasts 8 to 12 months (Oliveira, 2015), and from this moment onwards it reaches maturity, a period in which it becomes responsive to the inductive factors of flowering, which is a combination of short days and low temperatures (Cunha, 2005). Natural flowering is induced by low night temperatures, being accelerated at 20°C and inhibited at 30°C (Friend, 1981). According to Bartholomew and Malézieux (1994), plants subjected to a constant temperature of 25°C exhibit higher flowering rates in the eight-hour photoperiod, compared to the 16-hour photoperiod. Therefore, within a limit, the longer the night, the lower the temperature required.

The maximum flowering control period of the cultivar Pérola showed an average increase of three to four weeks in relation to ‘Vitória’. This result is possibly associated with characteristics that are intrinsic to the cultivars and that may have influenced the way the plant interacted with AVG. Our hypothesis is that the differences observed in the cell wall structure of these cultivars alter the absorption of the product, since AVG does not easily penetrate the plant cuticle (Kuan et al., 2005). Our hypothesis is supported by the studies of Aquije et al. (2011), who observed that the cultivar Pérola has a thinner and less rigid cell wall than ‘Vitória’, therefore, this fact may have favored the absorption of AVG in the plant, leading to an increase in the flowering control time.

Studies on the inhibitory effect of AVG in other pineapple cultivars corroborate the results of this research, indicating that higher concentrations lead to an increase in the flowering control time (Jímenez and Villalobos, 2019; Wang et al., 2007; Kuan et al., 2005; Lin et al., 2006). When higher concentrations are used (400-600 mg L^-1^), the plant takes longer to synthesize ACC synthase again (Rabie et al., 2013); however, this action varies depending on climatic factors and the frequency of application used. Lin et al. (2006) comment that the aviglycin limit for good control of natural induction is above 100 mg L^-1^ and not beyond 500 mg L^-1^. The studies carried out always seek to achieve AVG efficiency in controlling flowering using lower concentrations, to reduce the cost of the product in controlling natural induction.

Bartholomew et al. (2011) and Kuan et al. (2005) found that if applications are suspended before or started after the critical period, which is linked to the drop in temperature, flowering control is reduced, regardless of the concentration used. In our study, the result was similar, considering the control periods evaluated and the presence of inductive factors, since all applications covered the critical period (June and July). This result indicates that spraying carried out only during the period of greatest environmental pressure already promotes the desired result in flowering control, since the residual period of AVG is a maximum of two weeks (Wang et al., 2007) and, therefore, a greater number of sprays started outside the critical period would not lead to an increase in the floral inhibition time. Based on the results, a pineapple producer aware of this behavior could follow climate forecasts and begin applications before temperatures drop and end them when there is no longer any risk of the plants being naturally induced by the effects of climate conditions (Rabie et al., 2011).

The reduction in flowering control in P3 (June to July) may be associated with plant size and environmental conditions at the time of AVG application. Rabie et al. (2011) found that plants with an average mass of 1,106.00 g were more responsive to the occurrence of natural floral induction than those with 676 and 695 g. This behavior was also observed by Rabie et al. (2013). It is also reported that ethylene synthesis over time is recovered more quickly when sprays are performed during the critical period. In our study, plants sprayed in P3 had more time to develop vegetatively. In addition, this was the period of greatest environmental pressure, with reduced temperature (below 20°C) and reduced photoperiod, which may have contributed to the faster recovery of ethylene synthesis over time.

Jímenez and Villalobos (2019) in a study with pineapple ‘MD-2’, reported the occurrence of phytotoxicity caused by AVG, when using concentrations of 200, 400 and 800 mg L^-1^, performing 12 to 14 sprays, with a weekly interval. Wang et al. (2007), using the cultivar Tainon-17, also observed symptoms on the leaves with concentrations of 250, 375 and 500 mg L^-1^, performing four to five applications, with an interval of ten to 15 days. Both authors report that the plants recovered with barely evident symptoms, however, they do not make clear how long it took for this to happen.

The occurrence of phytotoxicity is not only associated with the concentration and period of application, but also with the sensitivity of the cultivar, since in other studies with similar concentrations, the occurrence of phytotoxicity was not reported. In the present study, a reduction in symptoms over time was also observed, however, according to the results presented, the percentage of leaves with symptoms four months after the end of the applications was still high, especially when the concentration of 400 mg L^-1^ of AVG was used.

The cultivar ‘Pérola’ showed a lower response to artificial floral induction, however, in the literature, it is not reported that AVG affects the sensitivity of pineapple plants to artificial floral induction, and the result found in ‘Pérola’ may be associated with the leaf damage caused by phytotoxicity in this cultivar, which also favored the severity of fusariosis, being greater at the concentration of 400 mg L^-1^. The occurrence of lesions in plant tissues allows the fungus to colonize the cells and the disease to occur (Ventura et al., 2009).

The reduction in chlorophyll levels is possibly associated with phytotoxicity, with one of the main symptoms of phytotoxicity being the occurrence of chlorosis in the leaves. According to studies by Dong et al. (2018), tea leaves (*Camelia sinensis* L.) with chlorosis presented chloroplasts with abnormal structural development, reduction in photosynthetic pigments and in the rate of net photosynthesis, when compared to completely green leaves. The chlorophyll content in the leaves is directly related to the potential for photosynthetic activity of plants, thus, the reduced capacity for photosynthesis also reduces carbon metabolism and, consequently, the supply of energy and carbon skeletons for plant growth and development (Garrido et al., 2023). The reduction observed in the vegetative development of plants may be a reflection of the result observed in photosynthetic pigments.

Another important factor not mentioned is the increase in chlorophyll b and carotenoids in relation to chlorophyll a. The possible cause is that the excessive accumulation of chlorophyll b in plants is associated with photo-oxidative stress (Yamasato et al., 2008), generated by the formation and accumulation of H_2_O_2_, which alters the plant’s gene expression and leads to cell lysis. This occurs because the transfer of energy between chlorophyll a in PSII was disturbed due to the exchange of chlorophyll a for chlorophyll b (Sakuraba et al., 2010; Tanaka and Tanaka, 2011). Likewise, the reduction in chlorophyll a levels may be associated with the action of AVG, which is not only linked to the blocking of the ACC synthase enzyme and ethylene biosynthesis, but also to other enzymes responsible for auxin biosynthesis and others linked to the metabolism of nitrogen, which is a fundamental constituent in the chlorophyll a molecule (Depaepe and Straeten, 2017).

The more significant reduction in reducing sugars in plants with a higher degree of phytotoxicity reinforces this relationship, since the main reducing sugars are glucose and fructose (Souza et al., 2013). The results of AST and starch indicate the recovery of the plant over time, since the collection for quantification of carbohydrates was carried out four months after the end of the spraying.

Despite the reduction observed through the application of AVG, only at a concentration of 400 mg L^-1^ were no fruits marketable in the domestic market produced, which is the predominant destination for Brazilian pineapple. According to Ceagesp (2003), fruits intended for consumption in natura must have a mass between 900 and 1,200 g. Therefore, at a concentration of 400 mg L^-1^, although it is the most efficient in floral inhibition, it results in fruits that do not meet commercial standards in the cultivars under study, and its use is not recommended. Matos and Reinhardt (2009) state that the ‘Pérola’ pineapple normally produces fruits with a mass of 1,000 to 2,000 g. On the other hand, in the ‘Vitória’ pineapple, the fruits can reach up to 1,500 g (Ventura et al., 2009). The values found in this study are close to the variation found for these two cultivars.

The effect of increasing AVG concentrations on reducing pineapple mass was also observed by other authors with the MD-2 cultivar. Bartholomew et al. (2011) did not observe a reduction in the fruit biomass of this cultivar at concentrations of 50, 75 and 100 mg L^-1^ applied seven to ten times at weekly intervals. However, in later studies by the same authors and at the same concentrations, in 14 and 17 applications, at weekly and biweekly intervals, a reduction in fruit mass was observed. The authors attributed this different result to the fact that in the second study, AVG applications were made late, during the early stages of infructescence development, resulting in deformation and a reduction in the number of marketable infructescences. Chen and Paull (2017), in a study also with the MD-2 cultivar, using concentrations of 100 and 200 mg L^-1^ of AVG, carrying out four to five applications, reported that spraying should begin before natural flowering occurs, to avoid deformation of the infructescences.

In our study, we did not associate the reduction in fruit mass with the application period, since according to the climate data, the sprayings were initiated before the natural induction period. The hypothesis that the occurrence of phytotoxicity in the plants influenced the development of the fruit is contradicted when considering the cultivars, since it was expected that the cultivar Pérola would present a greater reduction in mass since the phytotoxicity was more expressive in this cultivar.

By means of Normative Instruction/SARC No. 001 of February 1, 2002, the Brazilian Ministry of Agriculture, Livestock and Food Supply determined the Brazilian marketing standards for yellow and white pulp pineapples with at least 12° Brix. As observed in this study, the values obtained were within the ideal range for fresh marketing. The only exception are the fruits produced on plants of the Pérola cultivar, which did not receive the AVG, whose average value was 11° Brix.

The result found in the Pérola cultivar may be associated with the rainfall index recorded during harvest. Brasil et al. (2016), evaluating the physical-chemical quality of frozen fruits, reported that the occurrence of rain during the harvest period promotes the dilution of soluble solids. In our study, fruits from plants without AVG were harvested during the months of November to January 2019, a period in which the average rainfall observed was 259.66 mm, while in the rest of the period (February to July 2019) the average was 67.1 mm. Therefore, when considering fruit quality parameters, one should carefully observe the environmental conditions during fruit development, especially in the final stages of ripening.

One of the hypotheses for the increase in the soluble solids content due to the increase in the AVG concentration in the fruits of the Vitória cultivar is associated with the reduction in the mass of the fruits. In a study with this same cultivar, in the same location, Küster et al. (2018) found a negative correlation between the mass of the fruit and the content of soluble solids, indicating that heavier fruits tend to have a lower content of soluble solids. According to the data from our study, the reduction in fruit mass appears to have favored the concentration of soluble solids in the fruit.

The increase in acidity in the Pérola cultivar may have occurred due to temperature changes observed during fruit development. Sukporn et al. (2019) reported that fruits harvested with 25% ripe skin, in months with lower temperatures, showed an increase in titratable acidity. In our study, the harvest of fruits from plants sprayed with AVG occurred from March to July, a period in which an average minimum temperature of 19°C was observed. On the other hand, during the harvest of fruits from non-sprayed plants, the minimum temperatures were 21.4°C. This temperature difference may have influenced the titratable acidity of the cultivar.

The Ratio is considered a representative parameter of consumer acceptability because it expresses the proportion between sugars and acids, resulting in better characterization of the fruit flavor (Chitarra and Chitarra, 2005). The reduction in the Ratio in the Pérola cultivar was associated with a significant increase in acidity, indicating that this cultivar presents variations in flavor, depending on the AVG concentration and the time of harvest of the fruit.

## 5 Conclusion

In the cvs. Pérola and Vitória, floral induction of plants without the application of AGV (control) occurred mainly during the months of June and July.

AVG controlled the natural flowering of ‘Pérola’ and ‘Vitória’ pineapples. However, the Pérola cultivar was more responsive to natural flowering control, with a longer period of anthesis inhibition.

AVG applications must be carried out before unfavorable weather conditions occur for natural floral induction.

AVG caused phytotoxic effects on plants and the severity of symptoms increased with increasing concentration and when a greater number of applications were performed.

At a concentration of 400 mg L^-1^ of AVG, the cultivar Pérola was less responsive to artificial floral induction.

The concentration of 400 mg L^-1^ of AVG significantly reduces vegetative development and fruit biomass.

The absence of variation in the acidity of pineapple fruits of the Vitória cultivar was considered an advantage for scaling production, as it made it possible to produce fruits at different times without significant changes in quality.

Natural flowering control was 80% using 100 mg L^-1^ of AVG applied weekly from April onwards, extending throughout the induction period, without affecting fruit mass, which remained around 1.2 kg.

## Data Availability

The original contributions presented in the study are included in the article/supplementary material. Further inquiries can be directed to the corresponding author.
